# Southampton Arm Fracture Frailty and Sarcopenia Study (SAFFSS): a study protocol for the feasibility of assessing frailty and sarcopenia among older patients with an upper limb fracture

**DOI:** 10.1136/bmjopen-2019-031275

**Published:** 2019-08-15

**Authors:** Kinda Ibrahim, Mark Mullee, Guiqing Lily Yao, Shihua Zhu, Mark Baxter, Simon Tilly, Cynthia Russell, Helen C Roberts

**Affiliations:** 1 Academic Geriatric Medicine, Faculty of Medicine, Southampton University, Southampton, UK; 2 NIHR CLAHRC Wessex, University of Southampton, Southampton, UK; 3 Faculty of Medicine, University of Southampton, Southampton, UK; 4 Department of Health Sciences, University of Leicester, Leicester, UK; 5 Trauma and Orthopaedic Department, University Hospital Southampton NHS Foundation Trust, Southampton, UK; 6 Medicine for Older People, University Hospital Southampton, Southampton, UK

**Keywords:** frailty, sarcopenia, fracture, older people, feasibility

## Abstract

**Introduction:**

Falls are a major health problem for older people; 35% of people aged 65+ years fall every year, leading to fractures in 10%–15%. Upper limb fractures are often the first sign of osteoporosis and routine screening for osteoporosis is recommended by the National Institute for Health and Care Excellence to prevent subsequent hip fractures. However, both frailty and sarcopenia (muscle weakness) are associated with increased risk of falling and fracture but are not routinely identified in this group. The aim of this study is to evaluate the feasibility of assessing and managing frailty and sarcopenia among people aged 65+ years with an upper limb fracture.

**Methods and analysis:**

This study will be conducted in three fracture clinics in one acute trust in England. 100 people aged 65+ years with an upper arm fracture will be recruited and assessed using six validated frailty measures and two sarcopenia tools. The prevalence of the two conditions and the best tools to use will be determined. Those with either condition will be referred to geriatric clinical teams for comprehensive geriatric assessment (CGA). We will document the proportion who are referred for CGA and those who receive CGA. Other outcome measures including falls, fractures and healthcare resource use over 6 months will be collected. In-depth interviews with a purposive sample of patients who undergo the frailty and sarcopenia assessments and healthcare professionals in fracture clinics and geriatric services will be carried out to their acceptability of assessing frailty and sarcopenia in a busy environment.

**Ethics and dissemination:**

The study was given the relevant ethical approvals from NHS Research Ethics Committee (REC No: 18/NE/0377), the University Hospital Southampton NHS Foundation Trust, and the University of Southampton, Faculty of Medicine Ethics Committee and Research Governance Office. Findings will be published in scientific journals and presented to local, national and international conferences.

**Trial registration number:**

ISRCTN13848445

**Strengths and limitations of this study**

- This study will determine the feasibility of using six validated measures of assessing frailty and two for sarcopenia in older patients with fragility fracture in fracture clinics.
- Comparison of assessment methods for frailty and sarcopenia will enable the research team to recommend which tools are most suitable for use in both clinical practice and future research studies.
- We will determine the costs associated with identifying patients with frailty and sarcopenia in fracture clinics.
- We will report the prevalence of frailty and sarcopenia in this population. However, the small sample size may limit the generalisability of these data.
- Recruiting from three clinics in one UK hospital limits the generalisability and transferability of the findings.

## Introduction

Falls are a major health problem for older people, with a third of people aged 65+ years falling each year, increasing to 50% for those over 80 years of age.^1 2^ There are over 255 000 falls-related emergency hospital admissions/year in England alone.^1 2^ Upper limb fractures (‘fragility fractures’) often result from ‘low energy’ trauma (falling from a standing height) and are frequently the first sign of osteoporosis.^3^ Twenty-five per cent of patients with fragility fractures will suffer a subsequent fracture, often of the hip, within 10 years.^4 5^ Moreover, all fragility fractures are associated with increased 5-year mortality rates.^6^

Fragility fractures offer an excellent opportunity to identify osteoporosis early. In line with the National Institute for Health and Care Excellence (NICE) guidelines,^7^ fracture liaison services have been widely established in the UK with care pathways to assess and treat patients for osteoporosis to prevent hip fractures.^8^ It is estimated that by identifying these patients systematically, 25% of hip fractures (approximately 20 000 a year in the UK) can be prevented.^9^ NICE clinical guideline 161 recommends that future research studies focus on identifying the risk factors for falling that are most prevalent in the current UK older inpatient population to underpin the development of more effective and better targeted multifactorial assessments and interventions.^10^ Frailty and sarcopenia are recognised as risk factors for falls and fracture among patients with osteoporosis.^11 12^

Frailty is defined as a decline in multiple body systems, which increases an individual’s vulnerability to changes in their health or environment.^13^ In several studies, in both men and women, frailty was identified as an important risk factor for falls and fractures.^14–17^ A recent systematic review reported that frail and prefrail older patients were at higher risk of future fractures than patients without frailty.^18^ Among frail older patients, non-hip fragility fracture was reported to be associated with an almost threefold increase in the risk of a subsequent hip fracture within the next 2.5 years.^19^

There is no consensus on the best measure of frailty.^20^ The Fried Frailty Index, widely seen as a gold standard, is based on physical function alone. A similar self-reported tool—the Fatigue, Resistance, Ambulation, Illness and Loss of weight (FRAIL) scale—was recently developed^21^ as a simple tool for clinical practice but is reported to under-report frailty compared with the Fried Index.^22^ The Canadian Study of Health and Ageing Clinical Frailty Scale (CFS),^17^ increasingly being used in acute medical wards, uses health professionals’ clinical knowledge to categorise patients’ health and frailty against nine descriptions and images. The brief Preferred Reporting Items for Systematic Reviews and Meta-Analyses (PRISMA)-7 questionnaire is recommended by the British Geriatrics Society for use in clinical practice.^23^

The prevalence of frailty among hospitalised older people with hip fracture using the Fried Frailty Index has been estimated to be 50%.^24^ One UK study of 24 patients (aged 50+ years) with vertebral fragility fractures reported 71% of patients to be frail using the PRISMA-7 tool.^25^ The prevalence of frailty in patients with upper limb fracture is little explored, yet there is increasing recognition that frailty may be modifiable through multicomponent interventions including exercise and nutritional optimisation.^26^

Osteoporosis and sarcopenia (low muscle mass and strength) often coexist (*osteosarcopenia*) and both are associated with risk of disability, falls, frailty and fractures.^12 27^ In a study of 17 891 people from diverse ethnicities, participants with sarcopenia were twice as likely to have osteoporosis or reduced bone density.^28^ Several studies have reported a variable prevalence of sarcopenia among patients with hip fracture. An Italian study reported that 58%–64% of women and 95% of men (mean age 81 years) with hip fracture had sarcopenia.^29 30^ A Spanish study reported that 17.1% (12.4% in men, 18.3% in women) of older patients (mean age 85 years) with hip fracture had sarcopenia.^31^ A further study reported a higher incidence of sarcopenia among women (mean age 71 years) with fragility fractures than those without fractures (41.4% vs 19.3, p<0.018).^32^

Despite the increasing evidence for the association of fractures with frailty and sarcopenia, the two conditions remain under-recognised and therefore undertreated in patients with fragility fractures.^26 33^ Exercise and adequate nutrition, particularly with regard to vitamin D, calcium and protein, are key lifestyle approaches that can optimise bone, muscle and functional outcomes in older people, if they are individually tailored and appropriately prescribed.^27 34^

Early identification and treatment of sarcopenia has been suggested to be an important element in the future prevention of fractures.^35 36^ Comprehensive geriatric assessment (CGA)—a method of identifying and treating an older person’s medical, functional and psychosocial problems by multidisciplinary health and social care teams—can offer an opportunity to manage these age-related syndromes and improve health outcomes.^37^ A recent study has reported that CGA can help identify those with an increased risk of hip fractures allowing the implementation of prevention strategies.^38^ The aim of this study is to evaluate the feasibility of assessing people aged 65+ years, who attend fracture clinic with an upper limb fracture, for frailty and sarcopenia in addition to routine assessment for fracture risk due to osteoporosis. We will demonstrate whether it is practical and acceptable (to patients and staff) to assess for frailty and sarcopenia in a busy fracture clinic typical of those found in every general hospital, and then further manage these patients through geriatric clinical services. This feasibility study will inform a future randomised controlled trial.

## Methods and analysis

### Study objectives

1. Evaluate the feasibility of assessing patients for frailty and sarcopenia in a busy fracture clinic.
2. Evaluate the feasibility of using existing CGA care pathways following assessments for frailty and sarcopenia.
3. Determine the views of clinicians and patients on the acceptability of the assessments for frailty and sarcopenia and availability of current care pathways.
4. Decide which outcomes to use for a future randomised controlled trial.
5. Estimate the key resource usage of the intervention including those associated with referrals coming from the CGA process.

### Setting and participants

This is a mixed-methods feasibility study. Patients will be recruited from three fracture clinics in one acute hospital over 12 months (March 2019 to March 2020). Patients will be eligible to take part in the study if they are aged 65+ years, have a single arm fracture (wrist or upper arm), referred directly from Accident & Emergency, general practice, local minor injuries unit or other fracture clinics, able to give informed consent and not previously diagnosed with frailty and/or sarcopenia. Patients with pathological fractures, multiple or lower limb fractures, active cancer diagnosis or care home residents will be excluded.

### Intervention

Patients attending fracture clinics will be assessed for frailty and sarcopenia, in addition to their usual care. Patients identified as having either frailty or sarcopenia will be referred to existing local geriatric clinical services for specialist review including CGA. This is a multidisciplinary assessment and management of patients using health and social care pathways. These may include medical review of comorbidities and optimisation of medication; consideration of unmet needs in physical, cognitive and social domains; and referrals to other clinical, social care or voluntary services. These actions and referrals will be varied and focused on the individual patient’s recognised needs and their wishes.

### Sample size

Based on the precision of estimating the lowest reported incidence of frailty (7%) or sarcopenia (16%), that is, 7% we may determine the true incidence to within 7% with a sample size of 100 or within 8% for a sample size of 80 patients, with 95% CI. To allow for a 20% dropout rate we aim to recruit 100 participants to the study.

### Recruitment

Eligible patients identified by the fracture clinic team using electronic records will be sent an invitation letter and study information sheet prior to their clinic appointment, typically a few weeks after their fracture. Patients will have at least 24 hours to consider whether they would like to take part and the opportunity to discuss the study with a researcher on arrival at the clinic. Informed written consent will be obtained from all participants.

### Patient and public involvement

A researcher coapplicant (CR) has been involved throughout the design of this study. She will be supported by three to four other patient and public involvement (PPI) members. The PPI team has advised, for example, that participants should be invited to bring a companion; that travel and parking costs be covered; and dissemination should include non-medical publications. They have further suggested that the physical assessments of frailty and sarcopenia are conducted before the questionnaires to minimise the impact of participant fatigue. They reviewed recruitment material, patient information sheets, invitation letters and data collection tools to check readability and the order of questions and questionnaires, and participant assessment burden. They will be invited to attend all of the regular study management and steering group meetings and will review participant retention and any concerns, and research findings as well as aid dissemination.

### Feasibility outcomes

- *Feasibility of assessing frailty and sarcopenia among patients with upper limb fracture*: This will be determined by: (A) the percentage of people who are assessed by each tool (adequacy); (B) availability of required data and the number of missing data; (C) equipment (including cost, availability of functioning equipment and frequency of calibration); (D) the time for carrying out each assessment; and (E) acceptability of the tools by staff and patients (via interviews). This will determine the prevalence of frailty and sarcopenia among the study participants and which measures of assessing frailty and sarcopenia are most feasible in this population
- *Feasibility of using existing care pathways*: Patients identified as having either frailty or sarcopenia will be referred to local geriatric clinical services for specialist review as outlined in the intervention. The actions instigated from these assessments and referrals will be varied and individualised according to patient’s needs and wishes. These referrals may lead to additional attendance at outpatients, primary care or exercise classes, for example. We will report the number of patients identified to have frailty and/or sarcopenia who are referred to CGA, the number of those who receive CGA and the number and type of follow-up interventions.
- *Falls and fractures*: Participants will be asked to fill in a falls diary recording the date, suspected cause, location and the consequences of each fall. They will be contacted by telephone at 3 and 6 months after recruitment to collect self-assessed information on falls and fractures within the previous 3 months. This will establish whether quarterly data collection is suitable for the future trial.
- *Mortality*: Death rates within 6 months of recruitment will be collected from the hospital patient administration system (PAS).
- *Future outcome measures*: Baseline data on nutritional, physical and cognitive factors which may be associated with frailty and sarcopenia status will be collected. Quality of life and physical function will be measured at baseline, and 3 and 6 months after recruitment. The feasibility of using each of these assessments in this patient group will inform which instrument will be used in the future trial.
- *Healthcare resource use*: Testing the key resource usage of the intervention and downstream influence on service usage including those associated with referrals coming from the CGA process.
- *Quality of life*: Quality of life will be measured by EuroQual-5 dimensions-5 levels (EQ-5D-5L) and ICEpop CAPability measure for Older people (ICECAP-O). We will explore which instruments will be more sensitive in measuring quality of life changes for our study population and how often we should collect such information.

#### Data collection and assessment tools

At recruitment to the study, baseline data will be collected from patients in person by a researcher or will be abstracted from the patients’ clinical records (table 1). To minimise participant fatigue, physical assessments (eg, gait speed) will be assessed first. Then questionnaires will be administered varying the order of completion between participants. Repetitive questions or assessments will only be assessed once.

**Table 1:** Variables collected throughout the study

	Baseline T1	Follow-up	
		3 months T2	6 months T3
Age	X		
Gender			
Weight	X		
Height	X		
Marital status	X		
Usual residency	X		
Smoking	X		
Alcohol use	X		
Mobility	X		
Comorbidities	X		
Drugs	X	X	X
AMTS	X		
SNAQ	X		
Barthel index	X		X
Falls	X	X	X
Fractures	X	X	X
EQ-5D-5L	X	X	X
ICECAP-O	X	X	X
Gait speed	X		
Grip strength	X		
Muscle mass	X		
Rise from chair	X		
SARC-F	X		
Fried Frailty Phenotype	X		
FRAIL scale	X		
Clinical Frailty Scale	X		
PRISMA	X		
e-FI	X		
SOF scale	X		

AMTS, Abbreviated Mental Test Scale; EQ-5D-5L, EuroQual -5 dimensions-5 levels questionnaire; FRAIL, Fatigue, Resistance, Ambulation, Illnesses and Loss of weight; ICECAP-O, ICEpop CAPability measure for Older people; PRISMA, Preferred Reporting Items for Systematic Reviews and Meta-Analyses; SARC-F, 5-item questionnaire used as a screening tool for sarcopenia; SNAQ, Simplified Nutrition Appetite Questionnaire; SOF, Study of Osteoporotic Fracture; e-FI, electronic Frailty Index.

#### Baseline data

- Sociodemographic data: age, gender, usual residence, marital status, smoking, alcohol use, usual residency.
- Number of falls in the previous years, number and type of fractures.
- Comorbidities and medications.
- Appetite using the 4-item Simplified Nutritional Appetite Questionnaire.^39^
- Cognition will be assessed using the 10-item Abbreviated Mental Test Score.^40^
- Functional status: Barthel score will be collected from each participant at baseline.^41^
- Quality of life will be measured using EQ-5D-5L^42^ and ICECAP-O.^43^
- Frailty assessment tools: six tools will be used to assess frailty.
  1. Fried Frailty Index^44^: frailty measured by the presence of three or more of self-reported weight loss, exhaustion, low physical activity and measured slow walk time over 4 m (<0.8 m/s) and weak grip strength using a dynamometer according to a standardised protocol (maximum value will be recorded).^45^
  2. FRAIL scale: self-reported scale with five questions on fatigue, resistance, ambulation, illness and loss of weight.^21^
  3. Study of Osteoporotic Fracture (SOF) Criteria for Frailty assesses three components: weight loss, inability to do stand-up from a chair five times and self-reported lack of energy.^17^
  4. CFS: a subjective clinical evaluation by a healthcare professional matching the patient to one of nine descriptors, and figures in the domains of mobility, energy, physical activity and function.^46^
  5. PRISMA-7: a brief 7-item self-reported questionnaire.^47^
  6. electronic Frailty Index: derived from clinical data based on a count of 36 potential deficits.^48^
- Sarcopenia will be assessed using two methods: the 5-item questionnaire used as a screening tool for sarcopenia (SARC-F), and the European Working Group on Sarcopenia in Older People (EWGSOP) criteria.
  1. The SARC-F was recently developed and validated to screen for sarcopenia in clinical practice.^49^ It asks about strength, ambulation (walking independence), standing up from a chair, stair climbing and history of falls. This tool has been recommended to identify older people for further diagnostic evaluation for sarcopenia.^50 51^
  2. The EWGSOP criteria are the gold standard assessment for sarcopenia^52^ and have recently been updated.^53^ They use the presence of both low muscle function (grip strength and walking speed) and low muscle mass for the diagnosis of sarcopenia. Walking speed and grip strength will have already been measured in the assessment of frailty. Muscle mass will be measured using bioelectrical impedance, a simple, non-invasive technique, where electrodes at the wrist and ankle enable a small current (800 μA) to pass through the body at a range of frequencies and impedance is measured over seconds. This allows body composition to be estimated to derive skeletal muscle index (SMI, skeletal muscle mass divided by height squared) values.^54^ We will use published cut-off values for low SMI of <8.87 and <6.67 kg/m^2^ in men and women, respectively.^55^

#### Data collected at baseline and 3 and 6 months’ follow-up

Follow-up data will be collected by the researchers by telephone at 3 and 6 months after recruitment (table 1) and include:

- *Functional status*: Barthel score will be collected from each participant at 6 months’ follow-up.^41^
- *Resource usage*: Data collected at 3 and 6 months from different sources will inform which to use in the future trial. Sources include: a short questionnaire (modified version of the client service receipt inventory) on social and personal expenditure self-reported by participants at baseline, and 3 and 6 months which will be verified by extracting information about healthcare service usage from the hospital PAS and primary healthcare systems such as TPP SystmOne.
- *Quality of life*: EQ-5D-5L and ICECAP-O will be collected at 3 and 6 months.
- *Acceptability of the assessments for frailty and sarcopenia*: The views and experience of patients and staff of assessing frailty and sarcopenia in fracture clinics will be obtained using qualitative semistructured interviews. Semistructured interview guides have been codesigned with PPI researchers. Purposive sampling will be used to select: (1) patients (n=15–20) who completed frailty and sarcopenia assessments to include men and women with and without frailty and/or sarcopenia; (2) staff (n=5–8) including consultants and nurses involved in providing the care to patients in fracture clinics; and (3) staff (n=5–8) including consultant geriatricians, frailty practitioners and therapists from local geriatric clinical services. Interviews with patients will take place soon after enrolment to the study to maximise recall. Interviews will take place in the participants’ own homes and are anticipated to last for 30 min. Staff interviews will take place later in the recruitment period to capture how the additional assessments and referrals impacted on workload and service provision. All potential participants will receive an interview-specific information sheet and will be allowed at least 24 hours to decide whether to take part in the interview. Explicit written consent will be obtained from each participant prior to the interview. All interviews will be audio recorded using a digital recorder and will be transcribed for later analysis.

#### Study progress

Participant recruitment started in March 2019 and will finish in March 2020.

## Data analysis

### Quantitative data analysis

Data will be double entered onto an SPSS 22 database and participants will be identified only by a study ID number. In line with good practice,^56^ the feasibility outcomes will be reported descriptively and narratively. Numbers and characteristics of participants recruited will be summarised using appropriate descriptive statistics. The prevalence of frailty and sarcopenia will be calculated for each measure and reported with 95% CIs. Agreement between measures will be examined using Cohen’s kappa statistics. The feasibility and practicality of each frailty and sarcopenia measure will be examined according to the number of missing variables, the time required to complete them and their acceptability to staff and patients (from interview data).

The clinical outcomes, including falls and fractures as well as mortality rates, will be reported as descriptive statistics (counts (%), mean and median) with 95% CIs at each follow-up time point. Descriptive summaries of changes in clinical outcomes, physical function (Barthel score) and quality of life between baseline and 6 months’ follow-up will be presented.

### Qualitative data analysis

Interviews will be analysed using thematic analysis.^57^ A descriptive coding scheme will be developed from transcripts and based on participants’ perceptions and experiences. Two types of coding will be used: ‘open coding’ to locate themes followed by ‘focused coding’ to determine which themes repeat often and which represent unusual concerns. Two researchers (KI and HCR) will read and code the interviews separately to develop and agree on a list of themes that reflect the participants’ own views and experiences. Coding will proceed in an iterative way with detailed memos linking emergent themes. The perceptions and views of different participants will be compared using constant comparison. A software program (NVivo V.12)^58^ will be used to facilitate data analysis. To ensure data validation the following strategies will be used: data triangulation (collecting data from different groups of participants), multiple coding and respondent validation, which involves cross-checking interim research findings with respondents.

### Progression criteria

Criteria that will be used as guidance to assess the feasibility of progressing to a future randomised controlled trial^59–61^ will include:

- Prevalence: A high prevalence (≥80%) of either frailty or sarcopenia may indicate no need to assess, and therefore care pathways should assume patients have frailty and/or sarcopenia. A low prevalence (<10%) would indicate that assessment may not be appropriate in this population.
- Acceptability of assessing frailty and sarcopenia in the fracture clinic: <50% of patients with complete assessments of frailty or sarcopenia to indicate assessment is not feasible. The Trial Steering Committee will consider if protocol changes are practical to improve completion rates and therefore feasibility.
- Recruitment: At least 80% of patients to be recruited within 1 year of start of recruitment.

**Figure 1:**
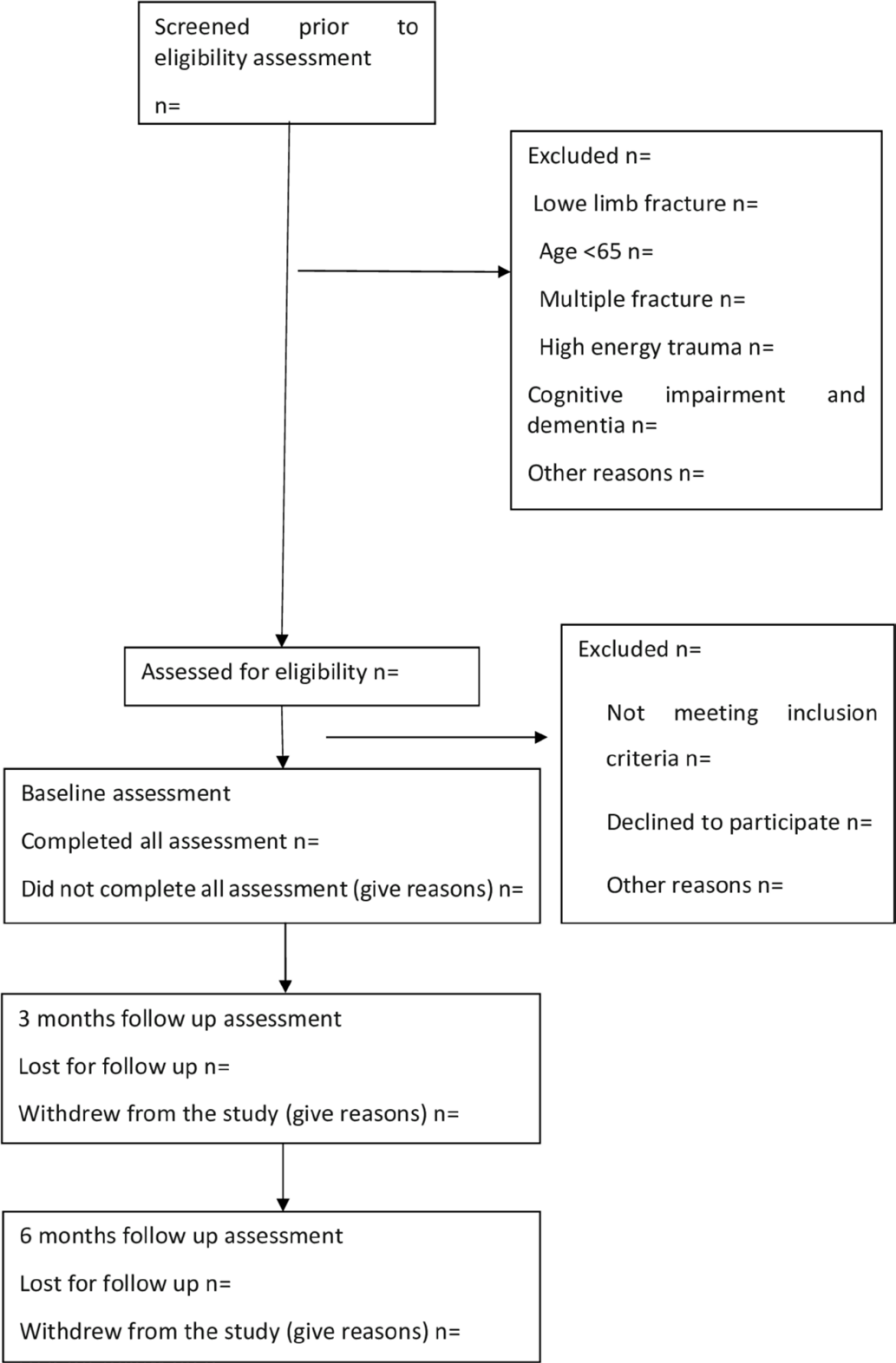
Study recruitment and follow-up diagram.

Not fulfilling the above criteria does not necessarily indicate failure but rather indicates changes to be made to protocol before proceeding to a definitive trial (figure 1).

Progression criteria will be reviewed by an independent Trial Steering Committee to determine if it is feasible to proceed to a definitive trial and if any protocol changes are required. Recruitment rates will be calculated by the number/proportion of eligible patients approached who agreed to participate. Adherence to protocols and completion rates will be examined by reporting the number/proportion of patients who complete the baseline and 6 months’ follow-up assessments.

### Health economic data analysis

Appropriate published national data: British National Formulary, National Reference Costs, Unit Costs of Health and Social Care (Personal Social Services Research Unit)^55^ and/or local (from hospital finance and trial manager) unit costs will be applied to itemised resource usage in calculating the total costs of the intervention and other NHS service usage. We will apply the UK tariff to translate the EQ-5D-5L and ICECAP-O questionnaire to utility scores.

Economic analyses in both costs and quality of life will be descriptive, reported as means with their SD. Correlation before utility scores with the main outcome (ie, number of falls prevented) will be analysed to see if there is evidence of sensitivity.

We will test the sensitivity and feasibility of both instruments with this patient group to inform which to use in a future trial.

### Ethics and dissemination

The study also obtained an ethical opinion for conduct by University of Southampton, Faculty of Medicine Ethics Committee and Research Governance Office. A consent form will be obtained by the research team from each participant prior to recruitment and another consent form will be obtained from those invited for interviews. Confidentiality and privacy will be ensured for all participants and the information gathered will only be used for scientific purposes. All participant information will be identified only by a study ID number. All data will be stored on a password-protected computer or in a locked filing cabinet in a secure office in our research unit and will be accessible only by the research team. In the analysis of results, data will be used anonymously and non-attributable to any individual. Our procedures for handling, processing, storing and destroying data are compliant with the Data Protection Act 2018. We will comply with Good Clinical Practice guidelines and the R&D measures of recording any serious adverse events (SAE) and reporting them immediately to the sponsor. Any incidents that result in hospitalisation, life threatening or death will be reported as SAEs immediately to the sponsor. The immediate reports will be followed promptly by detailed written reports. The immediate and follow-up reports will identify participants by their unique ID code that was assigned to the participant rather than their identifiable information such as name and/or address.

The study team will meet together monthly during the trial and will be responsible for ensuring high-quality delivery of the trial according to the agreed key milestones and deliverables. This will include early identification of potential problems, and implementing solutions to overcome them. A Trial Steering Group has been established to include the research team and external experts in the field who will meet every 6 months to oversee the study delivery and review the progression criteria. Amendments of any significant changes to the study design will be submitted to the relevant ethical committee to seek approval before implementation.

Findings from the study will be published in peer-reviewed high-impact journals and/or specialist open access journals. Research findings will be presented locally, nationally and internationally. We will work with our PPI team to further develop and implement the dissemination and engagement strategy including the development of reports and approaches to engage the community. We will disseminate the findings via social media (Facebook and Twitter), local groups and third sector, for example, Age UK and National Osteoporosis Society.

## Supplementary Material

Reviewer comments
