# Supplementary material for: Southampton Arm Fracture Frailty and Sarcopenia Study (SAFFSS): a study protocol for the feasibility of assessing frailty and sarcopenia among older patients with an upper limb fracture
Source: BMJ Open. 2019 Aug 15;9(8):e031275. doi: 10.1136/bmjopen-2019-031275 (PMC6701623; doi:10.1136/bmjopen-2019-031275)
Supplement: Reviewer comments [file bmjopen-2019-031275.reviewer_comments.pdf]

## PEER REVIEW HISTORY

BMJ Open publishes all reviews undertaken for accepted manuscripts. Reviewers are asked to complete a checklist review form (**http://bmjopen.bmj.com/site/about/resources/checklist.pdf**) and are provided with free text boxes to elaborate on their assessment. These free text comments are reproduced below.

## ARTICLE DETAILS

| | |
|---|---|
| **TITLE (PROVISIONAL)** | Southampton Arm Fracture Frailty and Sarcopenia Study (SAFFSS): a study protocol for the feasibility of assessing frailty and sarcopenia among older patients with an upper limb fracture |
| **AUTHORS** | Ibrahim, Kinda; Mullee, Mark; Yao, Guiqing; Zhu, Shihua; Baxter, Mark; Tilly, Simon; Russell, Cynthia; Roberts, Helen C |

This article was not externally reviewed at BMJ Open. Protocols that have been independently assessed prior to submission to BMJ Open are usually fast-tracked to publication on the grounds that further substantial changes will not be possible. This independent assessment will usually be external, independent review for both a substantial grant award from a non-commercial or government funder and ethics approval.
